# PML nuclear body disruption impairs DNA double-strand break sensing and repair in APL

**DOI:** 10.1038/cddis.2016.115

**Published:** 2016-07-28

**Authors:** A di Masi, D Cilli, F Berardinelli, A Talarico, I Pallavicini, R Pennisi, S Leone, A Antoccia, N I Noguera, F Lo-Coco, P Ascenzi, S Minucci, C Nervi

**Affiliations:** 1 Department of Science, Roma Tre University, Viale Guglielmo Marconi 446, Rome 00146, Italy; 2 Istituto Nazionale di Biostrutture e Biosistemi, Viale Medaglie d'Oro 305, Rome 00136, Italy; 3 IFOM-IEO Campus, Via Adamello 16, Milan 20139, Italy; 4 Department of Biomedicine and Prevention, University of Rome ‘Tor Vergata', Rome, Italy; 5 Neuro-Oncoematology Unit, Santa Lucia Foundation, Rome, Italy; 6 Department of Medico-Surgical Sciences and Biotechnologies, University of Rome ‘La Sapienza', Corso della Repubblica 79, Latina 04100, Italy

## Abstract

Proteins involved in DNA double-strand break (DSB) repair localize within the promyelocytic leukemia nuclear bodies (PML-NBs), whose disruption is at the root of the acute promyelocytic leukemia (APL) pathogenesis. All-*trans*-retinoic acid (RA) treatment induces PML-RAR*α* degradation, restores PML-NB functions, and causes terminal cell differentiation of APL blasts. However, the precise role of the APL-associated PML-RAR*α* oncoprotein and PML-NB integrity in the DSB response in APL leukemogenesis and tumor suppression is still lacking. Primary leukemia blasts isolated from APL patients showed high phosphorylation levels of H2AX (*γ*-H2AX), an initial DSBs sensor. By addressing the consequences of ionizing radiation (IR)-induced DSB response in primary APL blasts and RA-responsive and -resistant myeloid cell lines carrying endogenous or ectopically expressed PML-RAR*α*, before and after treatment with RA, we found that the disruption of PML-NBs is associated with delayed DSB response, as revealed by the impaired kinetic of disappearance of *γ*-H2AX and 53BP1 foci and activation of ATM and of its substrates H2AX, NBN, and CHK2. The disruption of PML-NB integrity by PML-RAR*α* also affects the IR-induced DSB response in a preleukemic mouse model of APL *in vivo*. We propose the oncoprotein-dependent PML-NB disruption and DDR impairment as relevant early events in APL tumorigenesis.

The DNA damage response (DDR) includes cell cycle arrest and transcriptional and post-translational activation of genes involved in DNA repair and triggering of apoptosis. Deficiencies in DDR are fundamental to the etiology of most human cancers.^1^

The DNA double-strand breaks (DSBs) arise from endogenous and exogenous sources, including replication errors, chemical mutagens, and ionizing radiation (IR).^2^ The sensing phase of the DSB response includes their recognition by the MRE11/RAD50/NBN complex, ATM protein activation, histone H2AX phosphorylation at Ser139 (*γ*-H2AX), MDC1, and 53BP1 recruitment. γ-H2AX is an initial DSB sensor for subsequent accumulation and post-translational modification (PTM) of signaling and repair proteins to form the so-called IR-induced foci (IRIF).^3, 4, 5, 6, 7, 8^ Finally, DSBs are removed through either the non-homologous end-joining (NHEJ) or the homologous recombination repair (HRR) pathways.^9^

Many proteins involved in the DDR localize within the promyelocytic leukemia (PML)-nuclear bodies (PML-NBs).^10, 11, 12, 13, 14, 15, 16^ PML-NBs are nuclear organelles implicated in cellular processes relevant to tumor suppression (e.g., PTM, DDR, transcriptional regulation, induction of apoptosis, and senescence).^16, 17, 18, 19, 20, 21^ PML-NBs increase in number and change their subnuclear distribution in response to DNA damage and represent structures where protein complexes are assembled, anchored, and/or post-translationally modified.^11, 13, 15, 16, 22^ PML also undergoes PTMs, SUMOylation representing one of the main modification necessary for the proper NB biogenesis and regulation of the cellular response to the DNA damage.^23^

Aberrant levels of the PML protein and loss of PML-NBS integrity were reported in acute promyelocytic leukemia (APL) and in other tumors.^24^ PML gene was originally cloned in APL leukemic cells carrying the t(15;17) detectable in over 95% of APLs. This translocation involves the PML and the retinoic acid receptor-*α* (RAR*α*) genes, and generates the PML-RAR*α* fusion gene.^25, 26, 27^ PML-RAR*α* acts as a transcriptional repressor antagonizing myeloid differentiation and promoting the self-renewal capacity of APL-initiating cells.^28, 29^ PML-RAR*α* also competitively inhibits the oligomerization of wild-type (WT) PML proteins, leading to the disruption of PML-NBs into nuclear ‘microspeckles'.^30^ In APL cells, all-*trans* retinoic acid (RA) treatment restores NB integrity, reverses PML-RAR*α*-mediated transcriptional inhibition, and induces PML-RAR*α* degradation and terminal cell differentiation.^29, 31, 32^

To date, it is yet unclear to what extent the DSBS repair is dependent upon PML and PML-NBS function. Here, the relationship existing between PML-NBS integrity and IR-induced DSBS sensing, signaling, and repair has been investigated in leukemic cells derived from APL individuals, myeloid cell lines expressing or not the PML-RAR*α* and in a PML-RAR*α* preleukemic mouse model *in vivo*. Data obtained show that the expression of PML-RAR*α* in myeloid cells causes basal damage and a defective DSBS response, highlighting the pivotal role of PML-NBs in coordinating and regulating the early and late events of DDR in APL. Overall, our results suggest that PML-RAR*α*-dependent PML-NBS disruption and DDR impairment are relevant early events in APL tumorigenesis.

## Results

### The integrity of PML-NBs is required for a proper DSBs rejoining

**Figure 1:**
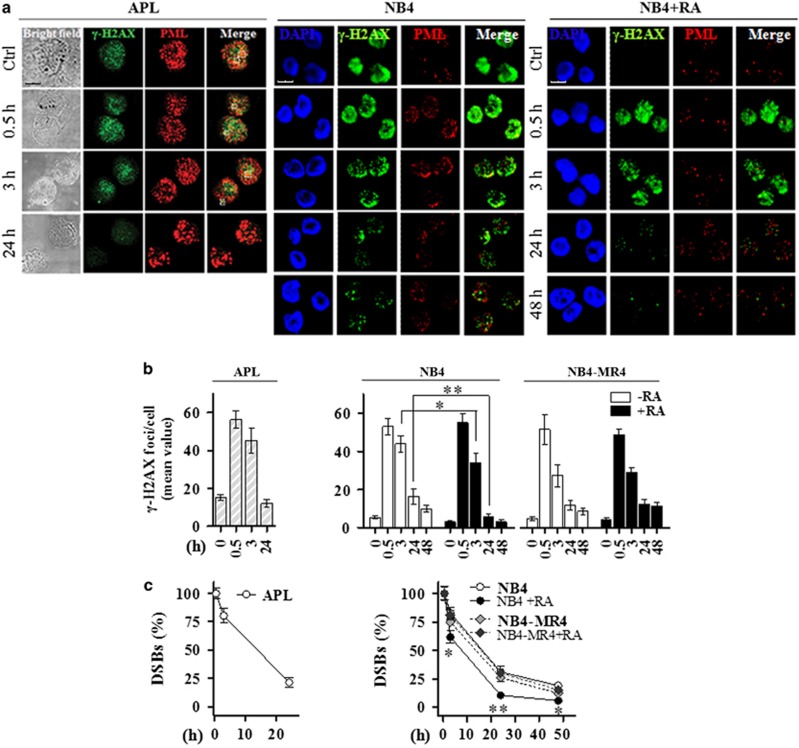
PML-NB integrity and *γ*-H2AX dephosphorylation kinetics. (**a**) Representative images of the double immunofluorescence analysis of *γ*-H2AX (Alexa Fluor 488, green fluorophore) and PML (Alexa Fluor 610, red fluorophore) foci in APL blasts untreated (Ctrl) and exposed to 1 Gy of X-rays and fixed after 0.5, 3, and 24 h (cell image: bright field; confocal microscopy images, magnification × 63), and NB4 cells treated or not with 1 *μ*M RA for 72 h, and then exposed to IR and fixed after 0.5, 3, 24, and 48 h (counterstain: DAPI; confocal microscopy images, magnification × 63). (**b**) Quantification of the mean number of *γ*-H2AX foci/cell and (**c**) analysis of the rejoining capability measured as percentage of DSBs in untreated and irradiated APL blasts derived from 3 different individuals, and NB4 and NB4-MR4 cells untreated or treated with 1 *μ*M RA for 72 h and then irradiated with 1 Gy. For NB4 and NB4-MR4 DSB rejoining graphs; * and ** indicate significant differences of RA-treated NB4 cells with respect to RA-untreated NB4 cells and NB4-MR4 cells. Mean values were derived from the analysis of 100 cells in 3 independent experiments±S.D. For DSBs, the mean number of *γ*-H2AX foci/cell measurable at 0.5 h after IR was taken as 100%. *P<0.05; **P<0.01. Confocal analysis was performed using the LCS Leica confocal microscope (Leica Microsystems, Heidelberg, Germany)

Double immunofluorescence staining with anti-PML and anti*-γ*-H2AX antibodies allowed the detection of disassembled PML-NBs and high number of *γ-*H2AX foci in primary blasts isolated at diagnosis from three APL patients, harboring the chromosomal translocation t(15;17) resulting in the PML-RAR*α* fusion product (also confirmed by RT-PCR, Figures 1a and b). Biological and clinical features of these APL cases are reported in Supplementary Table S1. Similar results were observed in the APL-derived NB4 cell line and in its RA-resistant derived subclone NB4-MR4 (Figure 1a and Supplementary Figure S1A).

The DSBs repair capability after 1 Gy of X-rays was evaluated as mean value of *γ*-H2AX foci/cell and as percentage of residual DSBs at each time point. In primary APL blasts, the number of *γ*-H2AX foci reached a peak after 0.5 h from IR, and slowly decreased after 3 h (∼45 foci/cell and 80% of unrepaired DSBs). A high number of unrepaired DSBs persisted after 24 h from IR, highlighting a delay in the DSBS repair in APL blasts (∼12 foci/cell and 24% of unrepaired DSBs) (Figures 1b and c).

Confocal microscopy also revealed a high number of *γ*-H2AX foci/cell in the untreated NB4 and RA-resistant NB4-MR4 cells (Figures 1 and b and Supplementary Figure S1). The peak in the mean value of *γ*-H2AX foci/cell was reached at 0.5 h from IR in both RA-untreated and RA-treated cells. A significant difference in the mean value of *γ*-H2AX foci/cell and in the percentage of residual DSBs was observed after 3 h from IR (~80% of unrepaired DSBs in NB4, NB4-MR4, and RA-treated NB4-MR4 cells; ~60% of unrepaired DSBs in RA-treated NB4 cells; *P*<0.05; Figures 1b and c). Similar to APL blasts, the DSB rejoining profile in NB4 cells showed that at 24–48 h from IR the number of persisting DSBs was significantly higher in NB4 cells and in NB4-MR4 cells, than in RA-treated NB4 cells (Figures 1b and c). In these cells PML-NBs were restored following PML-RAR*α* degradation and granulopoiesis was induced in a time-dependent and IR-independent manner, as revealed by an increased expression of he myeloid differentiation marker CD11b (Supplementary Figures S1B and C).

**Figure 2:**
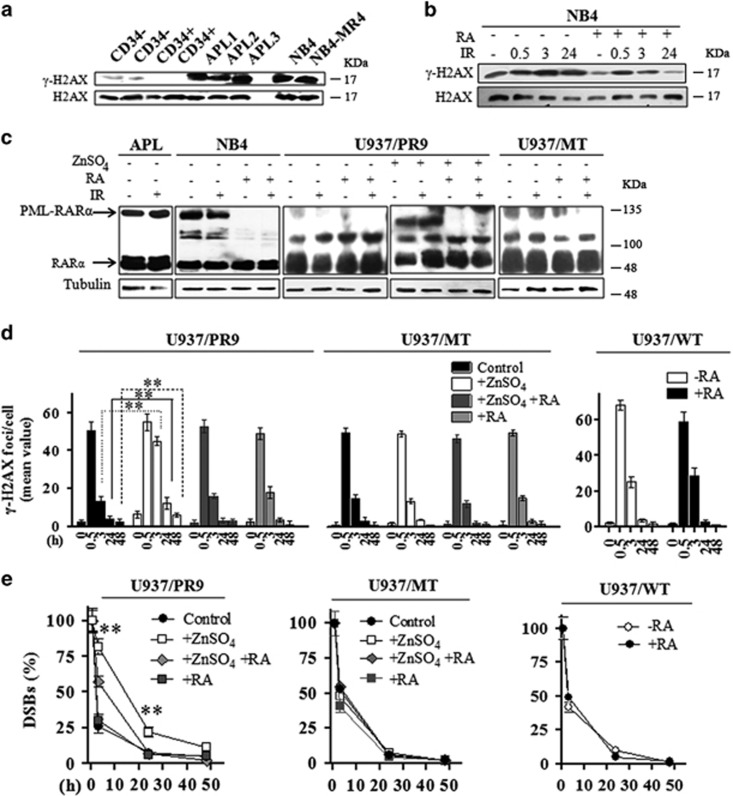
(**a**) Representative immunoblot analysis of H2AX and H2AX phosphorylation at the Ser139 residue in untreated human CD34− and CD34+ cells isolated from the peripheral blood of normal donors, in three APL patients, in NB4 and NB4-MR4 cells. (**b**) Representative immunoblot analysis of H2AX phosphorylation in NB4 cells treated or not with 1 *μ*M RA for 72 h and then irradiated with 1 Gy of X-rays and lysed after 0.5, 3, and 24 h. (**c**) Representative immunoblot analysis of RAR*α* and PML-RAR*α* expression levels in APL blasts, NB4, U937/PR9, and U937/MT cells exposed to IR and lysed after 0.5 h; before irradiation, NB4 cells were treated or not with 1 *μ*M RA for 72 h, whereas U937/PR9 cells were treated or not with 100 *μ*M ZnSO_4_ for 8 h and then with 1 *μ*M RA for 72 h, as indicated; filters were probed with anti-RAR*α* antibody, and tubulin was used as loading control. (**d**) Quantification of the mean number of *γ*-H2AX foci/cell and (**e**) analysis of the rejoining capability measured as percentage of DSBs in U937/PR9, U937/MT, and U937/WT cells treated or not with 100 *μ*M ZnSO_4_ for 8 h and/or with 1 *μ*M RA for 72 h, as indicated; cells were then exposed to IR and fixed at the indicated times. Mean values were derived from the analysis of 100 cells in three independent experiments±S.D. For DSBs, the mean number of *γ*-H2AX foci/cell measurable at 0.5 h after IR was taken as 100%. **P<0.01. For U937/PR9 DSB rejoining graph; **indicates significant differences of ZnSO_4_-treated U937/PR9 cells with respect to U937/PR9 control cells. Confocal analysis was performed using the LCS Leica confocal microscope (Leica Microsystems)

By immunoblot analysis we further observed that *γ*-H2AX was undetectable or barely detectable in human CD34+ hematopoietic progenitor cells (HPCs) and CD34− mononuclear cells (MNCs), respectively, whereas high levels of *γ*-H2AX were observed in PML-RARα-expressing samples from three APL patients, as well as in NB4 and NB4-MR4 cells (Figure 2a). RA treatment in NB4 cells decreased the levels of *γ*-H2AX when compared with untreated cells. Following IR, in NB4 cells the highest levels of *γ*-H2AX were detectable after 3 h, persisting until 24 h, whereas in RA-treated cells *γ*-H2AX levels reached a peak at 0.5 h and gradually decreased until 24 h (Figure 2b). IR treatment did not exert any effect on RAR*α* or PML-RAR*α* expression levels in primary APL blasts and NB4 cells (Figure 2c).

IR-treated U937/PR9 cells induced to express the PML-RAR*α* oncoprotein by ZnSO_4_ provided results similar to those observed in APL blasts and NB4 cells. U937/PR9+ZnSO_4_ cells displayed ~80%, 20%, and 10% of persisting DSBs after 3, 24, and 48 h from IR, respectively (Figures 2d and e). After RA treatment in U937/PR9+ZnSO_4_ cells, resulting in PML/RARα degradation and PML-NBS reformation, the percentage of persisting DSBs was 60%, 10%, and 2% after 3, 24, and 48 h, respectively. Interestingly, similar *γ*-H2AX foci/cell mean values and DSBS rejoining profiles were measurable, before or after treatment with ZnSO_4_ and/or RA, in myeloid cell lines expressing the WT PML protein such as U937/MT, U937/WT, HL60, and HL60-R, a RA-resistant subclone of HL60 (Figures 2d and e and Supplementary Figures S2A–C). Of note, the IR treatment did not exert any effect on RARα or PML-RARα expression levels in U937/PR9 and U937/MT cells (Figure 2c). This further suggested a role for the PML-NB disruption following PML-RAR*α* expression in the DSB rejoining proficiency of myeloid cells.

### The integrity of PML-NBs is required for the recruitment of 53BP1 to the DSBs

**Figure 3:**
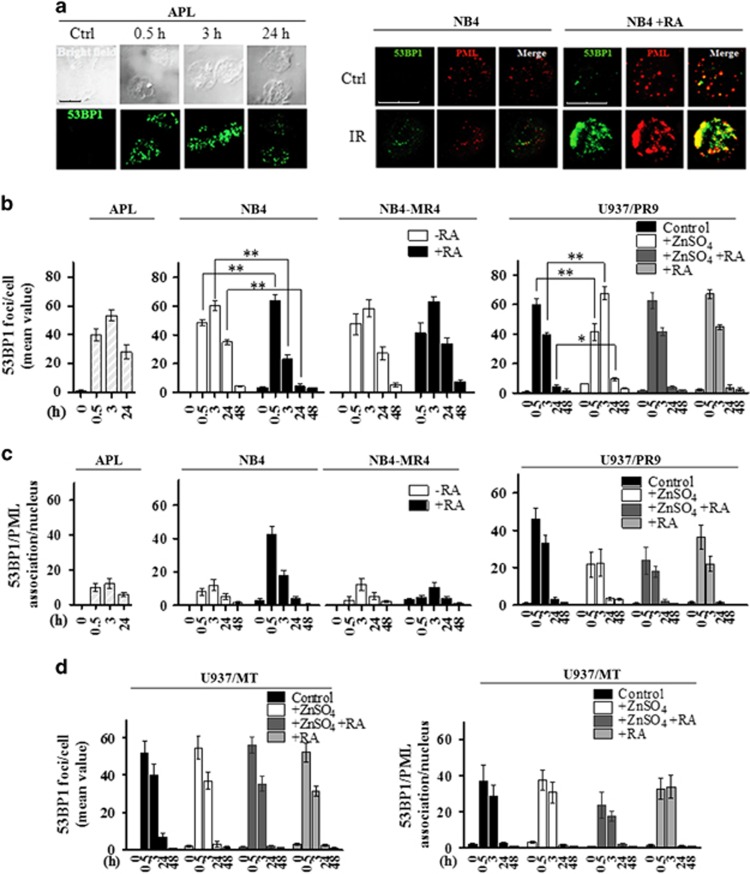
PML-NB integrity and 53BP1 recruitment to the DSBs. (**a**) Representative images of 53BP1 foci disappearance in APL blasts untreated (Ctrl) and exposed to 1 Gy and fixed after 0.5, 3, and 24 h, and in RA-untreated (NB4) and RA-treated (NB4+RA) cells non-irradiated (Ctrl) and exposed to IR and fixed after 0.5 h (cell image: bright field; confocal microscopy images, magnification × 63; 53BP1 (Alexa Fluor 488, green fluorophore); PML (Alexa Fluor 610, red fluorophore)). (**b**) Quantification of 53BP1 foci/cell, reported as the mean value of 53BP1 foci in APL blasts, NB4 and NB4-MR4 cells untreated or treated with 1 *μ*M RA for 72 h, and U937/PR9 cells either untreated or exposed for 8 h to 100 *μ*M ZnSO_4_ and then treated or not with 1 *μ*M RA for 72 h. After ZnSO_4_ and/or RA treatment, cells were irradiated with 1 Gy and fixed at the indicated time points. (**c**) Quantification of 53BP1 foci association events with PML per nucleus in APL blasts, NB4, NB4-MR4, and U937/PR9 cells. (**d**) Quantification of 53BP1 foci/cell and 53BP1 foci association events with PML per nucleus in U937/MT cells. Before irradiation, U937/MT cells were either untreated or exposed for 8 h to 100 *μ*M ZnSO_4_ and then treated or not with 1 *μ*M RA for 72 h. Mean values were derived from the analysis of 100 cells in three independent experiments±S.D. *P<0.05; **P<0.01. Confocal analysis was performed using the LCS Leica confocal microscope (Leica Microsystems)

53BP1 accumulates within the PML-NBs and is recruited into IRIF after DSBS induction, promoting the activation of the repair signaling.^33^ Therefore, we studied the DSB kinetics by counting the number of 53BP1 foci in primary APL cells and NB4 and NB4-MR4 cells after 0.5, 3, and 24 h from irradiation with 1 Gy. We found that PML-NBS integrity is required for 53BP1 localization into the nuclei and for 53BP1 foci formation after DSBS induction. In fact, 53BP1 was barely detectable in non-irradiated APL blasts and NB4 cells, probably because of a weak basal expression of 53BP1 or of its pan-nuclear dispersion into the disassembled PML-NBs. On the contrary, 53BP1 colocalized with PML within the restored PML-NBs following RA treatment of NB4 cells (Figure 3a). After IR-induced damage, the 53BP1 foci number and colocalization with PML was significantly lower in RA-untreated APL blasts and NB4 and NB4-MR4 cells compared with RA-treated NB4 cells (Figures 3a–c). Thus, restoration of the 53BP1 foci within the reformed PML-NBs may occur as a consequence of the RA-induced PML-RAR*α* degradation.

We evaluated the DSBs rejoining efficiency in cells expressing the PML-RAR*α* oncoprotein and in cells expressing the WT PML by counting the number of 53BP1 foci/cell in untreated and irradiated cells (Figures 3b and d and Supplementary Figure S2D). After 0.5 h from IR, APL blasts and NB4 cells showed mean numbers of 53BP1 foci/cell lower than those calculated in cells expressing WT PML or in cells where PML-RAR*α* was degraded by RA. After 3 h from IR, APL blasts and NB4 cells showed a number of 53BP1 foci significantly higher than that measurable in cells expressing WT PML or where the PML-RAR*α* was degraded by RA. Finally, after 24 and 48 h from IR, the residual mean value of 53BP1 foci/cell scored in cells expressing the PML-RAR*α* fusion protein was significantly higher compared with those expressing WT PML (Figures 3b and d and Supplementary Figure S2D).

### PML-RAR*α* does not increase chromosomal in stability, S phase, and cell death

**Figure 4:**
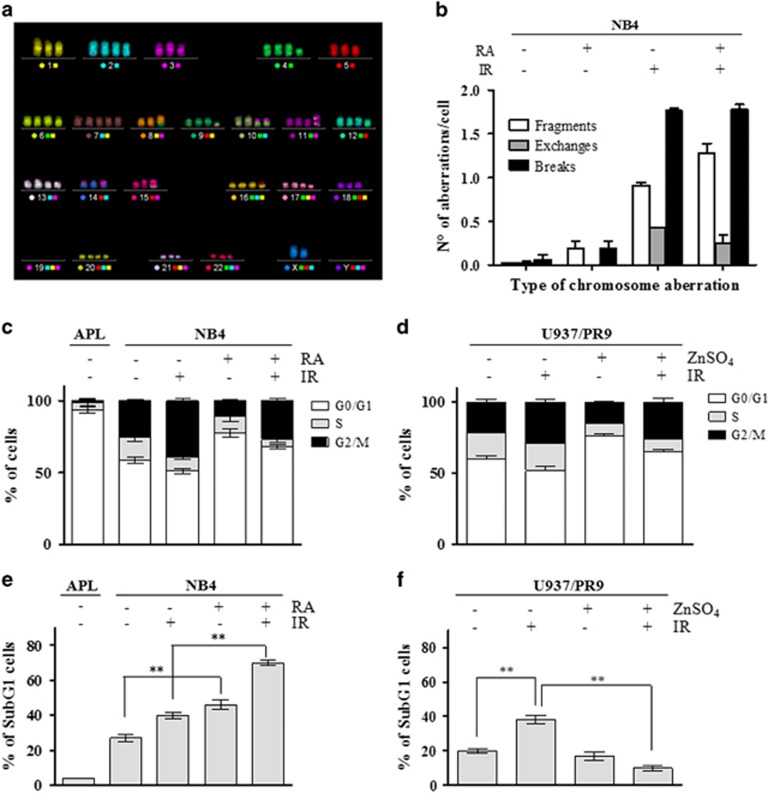
Chromosomal damage, cell cycle distribution, and apoptosis in PML-RAR*α*-expressing cells. (**a**) Representative image of an mFISH-stained NB4 karyotype. Karyotype was established considering conserved translocations that appear in >90% of the cells analyzed in controls. Metaphases were captured with the Axio Imager M1 microscope (Carl Zeiss, Oberkochen, Germany). Karyotyping and cytogenetic analysis of each single chromosome was performed by the ISIS software. (**b**) Frequency per cell of chromosomal aberrations (i.e., fragments, exchanges, and breaks) after irradiation with 1 Gy in NB4 cells untreated or exposed to 1 *μ*M RA for 72 h. (**c**) Cell cycle distribution of APL blasts and of NB4 cells untreated or treated with 1 *μ*M RA for 72 h and then irradiated and fixed after 24 h. (**d**) Cell cycle distribution of U937/PR9 cells untreated or treated with 100 *μ*M ZnSO_4_ for 8 h to induce PML-RAR*α* expression and then irradiated and fixed after 24 h. (**e**) Sub-G1 population analyzed by flow cytometry in APL blasts and in NB4 cells untreated or treated with 1 *μ*M RA for 72 h, and then irradiated and fixed after 24 h. (**f**) Sub- G1 population analyzed by flow cytometry in U937/PR9 cells untreated or treated with 100 *μ*M ZnSO_4_ for 8 h, and then irradiated and fixed after 24 h. Mean values were derived from three independent experiments±S.D. **P<0.01

To evaluate whether the residual *γ*-H2AX and 53BP1 foci/cell observed after 24 and 48 h from IR in APL blasts and PML-RAR*α*-expressing cells may correlate with chromosome instability, the multicolor FISH (mFISH) analysis was performed in NB4 cells, either untreated or exposed to RA, in combination with exposure to 1 Gy. The karyotype of NB4 was established considering conserved translocations that appear in >90% of the cells analyzed in controls. Karyotype was hypotetraploid and can be summarized as follows: 80 XX, −X, −X, −1, −3, −5, +7, −8, −14, −15, −18, −19, −19, −19, −19, −21, −22, t(8′–9), (9′–8), t(10–19), t(10–19), t(14–19), t(15′–17), t(16–5) t(17′–15), t(17′–15) (Figures 4a and b). The analysis of chromosomal exchanges in each sample was performed ignoring basal translocations observed in control cells. Results obtained indicated that RA alone did not exert any effect on the frequency of chromosome aberrations over control values and did not modify IR-induced break frequency (Figure 4b). Although break frequency remain unchanged, RA pretreatment reduced the frequency of IR-induced chromosome exchanges (from 2 to 0.9 exchanges/cell in NB4 control and RA-treated irradiated samples, respectively), whereas it increased the frequency of chromosomal excess fragments (from 1.8 to 3.9 fragments/cells in NB4 control and RA-treated irradiated samples, respectively) (Figure 4b). The only difference observed in RA-treated *versus* untreated NB4 samples was in terms of quality of the IR-induced chromosome damage rather than in terms of quantity (total breaks). Unfortunately, cytogenetic analysis could not be performed on primary APL samples because of the very slow proliferation rate of blasts isolated from the peripheral blood that are blocked in the G0/G1 phase of the cell cycle (Figure 4c).

As H2AX is phosphorylated either during the S phase of the cell cycle or as a consequence of cell death induction,^34^ we analyzed the cell cycle profile and the sub-G1 population of APL blasts and myeloid cell lines. In APL blasts, >90% of cells were in G1, 2% in S and 8% in G2/M phases of the cell cycle (Figure 4c), whereas the sub-G1 population was ~4% (Figure 4e). Cell cycle analysis in NB4 and U937/PR9 cells treated or not with RA and ZnSO_4_, respectively, untreated or irradiated with 1 Gy, showed that the high number of *γ*-H2AX foci/cell scored in PML-RAR*α*-expressing cells did not correlate with the percentage of cells in the S phase (Figures 4c and d). Moreover, in NB4 and U937/PR9 cells, no association was observed between the number of *γ*-H2AX foci/cell and cell death, both in basal conditions and after 24 h from IR exposure (Figures 4e and f). Overall, data obtained indicated that the high basal levels of *γ*-H2AX foci observed in APL blasts and PML-RAR*α*-expressing cells are not associated with the cell cycle S-phase or with cell death.

### The integrity of PML-NBs is required for ATM activation

**Figure 5:**
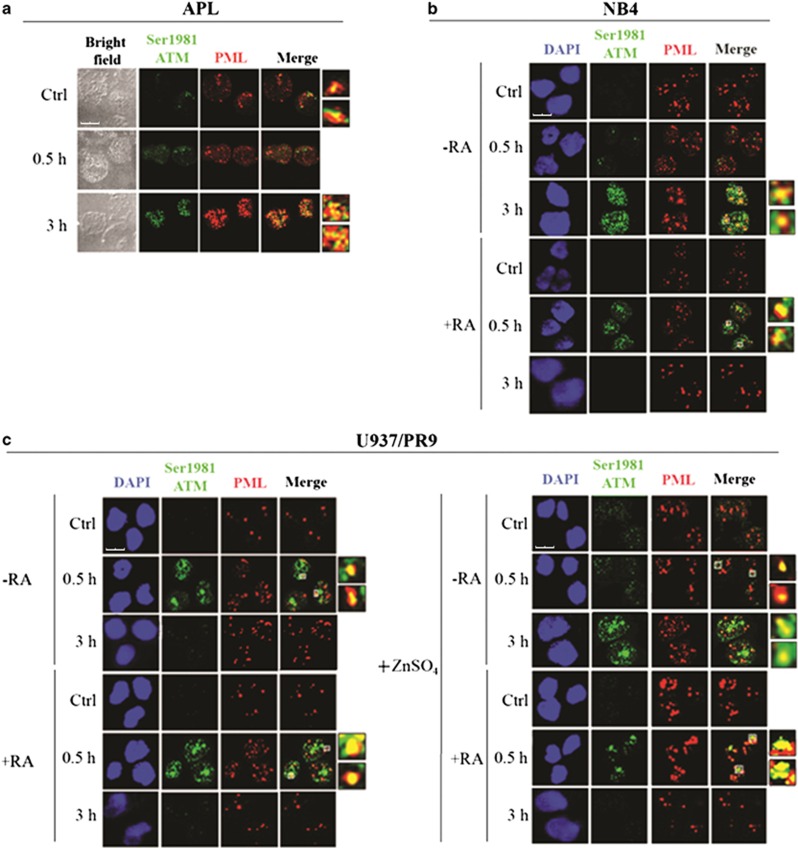
PML-NB integrity and ATM activation. Double immunofluorescence of pSer1981-ATM (Alexa Fluor 488, green fluorophore) and PML (Alexa Fluor 610, red fluorophore) foci performed in cells untreated or irradiated with 1 Gy and fixed after 0.5 and 3 h. Representative images of the analysis performed in (**a**) APL blasts (cell image: bright field), (**b**) NB4 cells untreated or treated with 1 *μ*M RA for 72 h previous to IR (counterstain: DAPI), and (**c**) U937/PR9 cells either untreated or exposed for 8 h to 100 *μ*M ZnSO_4_ and then untreated or treated with 1 *μ*M RA for 72 h previous to IR (counterstain: DAPI). Some colocalization signals have been highlighted and marked within the cell by a white square. Confocal microscopy images, magnification × 63; LCS Leica confocal microscope (Leica Microsystems)

ATM kinase, a critical regulator of the DDR, is rapidly phosphorylated at the Ser1981 residue in response to IR, and its active form in turn phosphorylates H2AX at Ser139, as well as several other component of the DDR (e.g., NBN at Ser 343 and CHK2 at Thr68).^2^ We evaluated the role of PML-NBS integrity in ATM phosphorylation at Ser1981 and activation in response to IR. ATM resulted poorly activated in non-irradiated PML-RAR*α*-expressing APL blasts, its Ser1981-phosphorylated form colocalizing with PML at 3 h from the irradiation with 1 Gy (Figure 5a). Similar to primary APL blasts, pSer1981-ATM foci were barely detectable in non-irradiated NB4 and U937/PR9+ZnSO_4_ cells (Figures 5b and c). In PML-RAR*α*-expressing cells, ATM activation and colocalization with PML was well detectable after 3 h from IR, whereas in cells expressing the WT PML (i.e., U937/WT and U937/PR9) an accumulation of pSer1981-ATM in PML foci was already visible after 0.5 h from IR (Figure 5c and Supplementary Figure S3A). Interestingly, RA treatment in both NB4 cells and Zn-induced U937/PR9 restored the pSer1981-ATM/PML colocalization pattern into PML-NBs after 0.5 h from IR (Figures 5b and c).

### The integrity of PML-NBs is required for DSBS signal transduction

**Figure 6:**
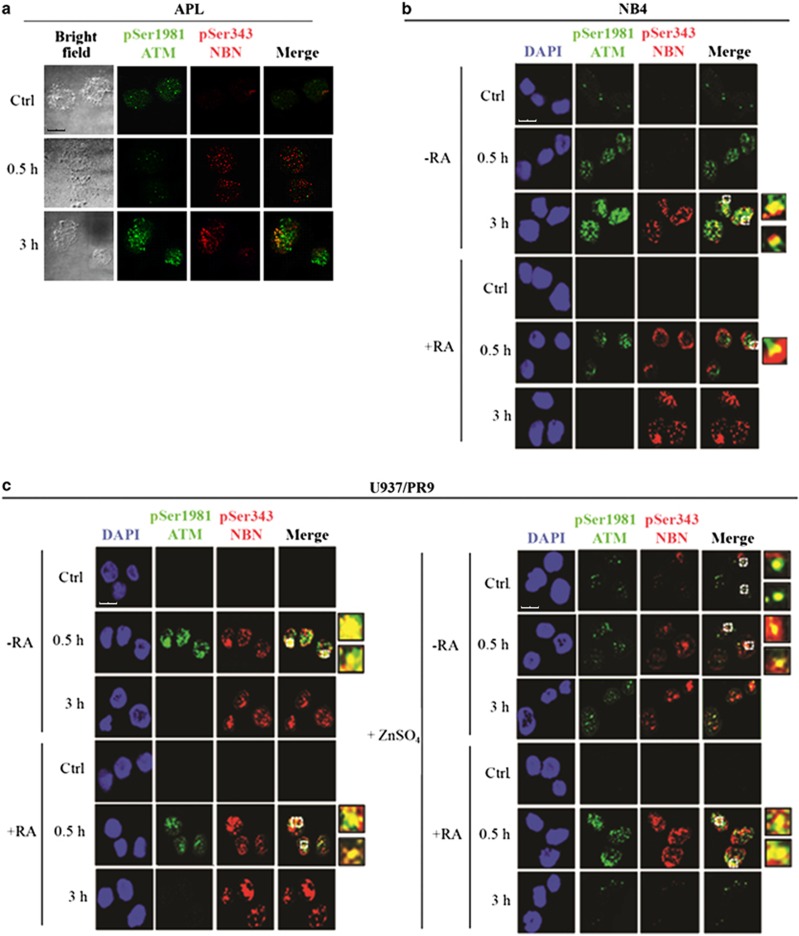
PML-NB integrity and ATM-dependent NBN phosphorylation. Double immunofluorescence of pSer1981-ATM (Alexa Fluor 488, green fluorophore) and pSer343-NBN (Alexa Fluor 610, red fluorophore) foci performed in cells untreated or irradiated with 1 Gy and fixed after 0.5 and 3 h. Representative images of the analysis performed in (**a**) APL blasts (cell image: bright field), (**b**) NB4 cells untreated or treated with 1 *μ*M RA for 72 h previous to IR (counterstain: DAPI), and (**c**) U937/PR9 cells either untreated or exposed for 8 h to 100 *μ*M ZnSO_4_ and then treated or not with 1 *μ*M RA for 72 h previous to IR (counterstain: DAPI). Some colocalization signals have been highlighted and marked within the cell by a white square. Confocal microscopy images, magnification × 63; LCS Leica confocal microscope (Leica Microsystems)

Co-immunofluorescence experiments with anti-pSer1981-ATM and anti-pSer343-NBN antibodies indicated that ATM and NBN phosphorylation were significantly induced after 3 h from irradiation with 1 Gy in PML-RAR*α*-expressing cells (Figure 6). In PML-RAR*α* cells treated with RA (NB4+RA and Zn-induced U937/PR9+RA) and in cells expressing the WT PML (U937/WT and U937/PR9 cells), a high number of pSer1981-ATM foci, colocalizing with pSer343-NBN, was visible at 0.5 h from IR; at 3 h from IR, pSer1981-ATM foci disappeared whereas NBN foci persisted (Figures 6b and c and Supplementary Figure S3B). These findings are in agreement with the proposed function of NBN in several phases of the DDR.^35, 36^

**Figure 7:**
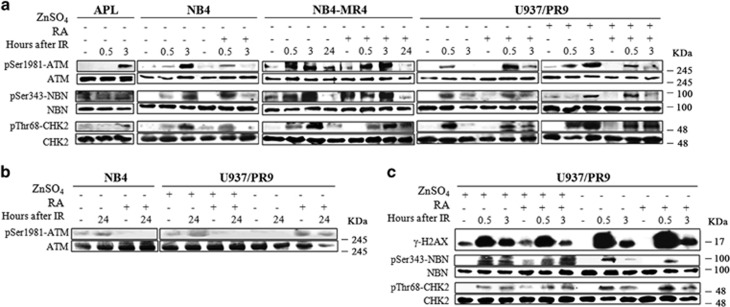
Phosphorylation of ATM kinase and its substrates in PML-RAR*α*-expressing cells. (**a**) Immunoblot analysis of pSer1981-ATM, pSer343-NBN, and pThr68-CHK2 phosphorylation in APL blasts, NB4, NB4-MR4, and U937/PR9 cells. Previous to IR, NB4 and NB4-MR4 cells were either untreated or exposed to 1 *μ*M RA for 72 h, whereas U937/PR9 cells were either untreated or exposed for 8 h to 100 *μ*M ZnSO_4_, and then treated or not with 1 *μ*M RA for 72 h. Cells were then exposed to 1 Gy of X-rays and lysed after 0.5, 3, and 24 h. (**b**) Immunoblot analysis of ATM phosphorylation at the Ser1981 residue after 24 h from IR in NB4 and U937/PR9 cells. (**c**) U937/PR9 cells were first treated with 100 *μ*M of ZnSO_4_, then exposed to 10 *μ*g/ml cycloheximide, and finally irradiated with 1 Gy and lysed after 0.5 h. Immunoblots were performed using anti-*γ*-H2AX, anti-pSer343-NBN, anti-NBN, anti-pThr68-CHK2, and anti-CHK2 antibodies. Blots presented are exemplificative of the results obtained from two independent experiments

Immunoblot analysis further showed that in PML-RAR*α*-expressing cells, ATM, NBN, and CHK2 proteins were phosphorylated in response to IR although with a delayed kinetics (Figure 7a). Indeed, the phosphorylation signal of ATM, NBN, and CHK2 reached a peak after 3 h from IR, ATM phosphorylation being visible up to 24 h from irradiation (Figures 7a and b), whereas in cells expressing WT PML, the ATM, NBN, and CHK2 proteins were phosphorylated after 0.5 h from IR, the phosphorylation signal being strongly reduced after 3 and 24 h (Figures 7a and b and Supplementary Figure S4A). These data indicate that PML-NBS integrity is necessary for a correct ATM–CHK2 axis activation.^22^ Furthermore, immunoblot analysis showed that H2AX, NBN, and CHK2 expression and phosphorylation levels were not affected in U937/PR9 cells treated or not with ZnSO_4_, then exposed to cycloheximide and finally irradiated, thus suggesting that *de novo* protein synthesis is not involved in the DDR signaling defects detected in APL cells (Figure 7c).

### Defective DSBS sensing and repair in a preleukemic mouse model of APL

**Figure 8:**
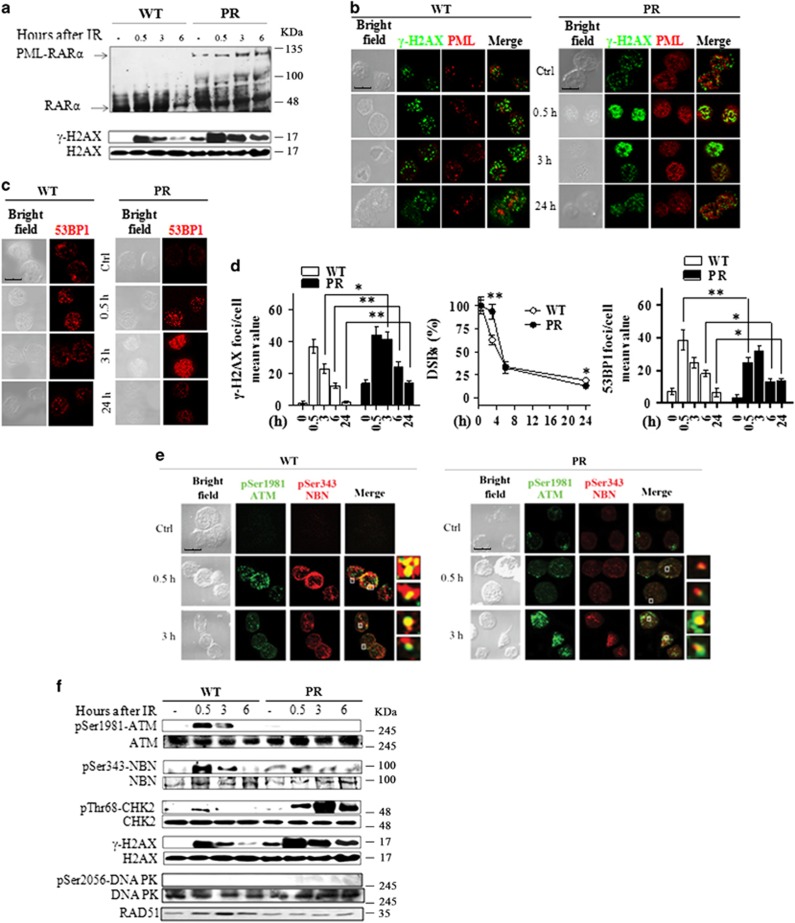
*In vivo* validation of the DDR in the preleukemic mouse model of APL. WT and preleukemic mice knock-in for PML-RAR*α* (PR) were irradiated with 5.5 Gy of X-rays and sacrificied after 0.5, 3, 6, and 24 h. Lin− cells were isolated from the bone marrow of three pooled mice. (**a**) Immunoblot analysis of RAR*α* and PML-RARα expression, and of H2AX phosphorylation at Ser139 residue, in untreated and irradiated WT and PR mice. (**b**) Representative images of the double immunofluorescence analysis of *γ*-H2AX (Alexa Fluor 488, green fluorophore) and PML (Alexa Fluor 610, red fluorophore) foci in untreated and irradiated WT and PR mice. (**c**) Representative images of the 53BP1 foci in untreated and irradiated WT and PR mice. (**d**) The DSBs rejoining analysis was reported as the mean value of *γ-*H2AX foci/cell and as the percentage of residual DSBs in untreated and irradiated WT and PR mice. The DSBS repair was also analyzed by counting the number of 53BP1 foci/cell in untreated and irradiated WT and PR mice. Mean values were derived from the analysis of 100 cells from three independent experiments±S.D. *P<0.05, **P<0.01. (**e**) Representative images of the double immunofluorescence analysis of pSer1981-ATM (Alexa Fluor 488, green fluorophore) and pSer343-NBN (Alexa Fluor 610, red fluorophore) foci in WT and PR mice. (**f**) Immunoblot analysis of ATM phosphorylation at the Ser1981 residue, NBN phosphorylation at Ser343, CHK2 phosphorylation at Thr68, DNA-PK phosphorylation at Ser2056, and RAD51 expression. Cell image: bright field; confocal microscopy images, magnification × 63; LCS Leica confocal microscope (Leica Microsystems)

To evaluate the role of PML-RAR*α* expression in the DSBS sensing and signaling *in vivo*, we used the preleukemic model of APL (i.e., PR mice).^37^ In this model, the disease develops after a long latency, suggesting that additional alterations cooperate with PML-RAR*α* for leukemia development. We confirmed that in Lin^−^ HPCs from these mice the expression levels of RAR*α* and PML-RAR*α* were not affected by IR. Of note, the levels of *γ*-H2AX were higher in untreated PR as compared with WT mice (Figures 8a and b).

PR mice HPCs showed a microsplecked nuclear pattern of PML-NBs whose number increased after irradiation with 5.5 Gy (Figure 8b). Kinetics of both *γ*-H2AX dephosphorylation and 53BP1 recruitment at the DSBs were delayed in preleukemic PR mice as compared with WT animals, with the persistence of significantly higher levels of unrepaired damage after 24 h from IR (*P*<0.05; Figures 8b–d). In addition, the ATM activation, as well as the phosphorylation of its substrates NBN and CHK2, was delayed in PR mice as compared with WT mice (Figures 8e and f). To analyze the DSBS repair pathway active in WT and PR mice, we looked at the status of the DNA-PK and RAD51 that play key roles in the NHEJ repair and HRR, respectively.^9^ Results showed that DNA-PK was not phosphorylated following IR either in WT or PR mice, whereas RAD51 was induced at 3 h in irradiated WT mice (Figure 8f). These results suggested that PML-RAR*α* expression impairs the IR-induced activation of HRR *in vivo*.

## Discussion

Disruption of PML-NBs by PML-RAR*α* is a hallmark of APL, a model disease to understand leukemogenic pathways directed by an oncoprotein.^24, 25, 26, 27, 28, 29, 30, 31, 32^ Here, we report that PML-NBs play a key role in regulating early and late steps of IR-induced DSBs sensing, signaling, and repair in myeloid cells *in vitro* and *in vivo*. These events may depend on the cellular context as PML depletion/overexpression and PML-RAR*α* ectopic expression in non-hematopoietic cells and solid tumors only impairs the late phases of HRR.^16^

Results obtained in a PML-RAR*α* preleukemic knock-in mouse model strongly suggest that the PML-RARα-induced PML-NBs disruption is associated with DDR impairment and represents an early event in APL pathogenesis. Evidence obtained in myeloid cells expressing or not the PML-RAR*α* and in RA-resistant myeloid cells lines suggested that RA-induced reformation of PML-NBs and oncoprotein degradation, two events contributing to the therapeutic effect of RA in APL, is tightly correlated with the restoration of DDR in APL cells. Moreover, our results point to a direct involvement of RA binding to the endogenous PML-RAR*α* in the restoration of PML-NB integrity and DDR.

In agreement with previous findings obtained in APL cell lines and in hematopoietic precursors from mice expressing PML-RAR*α*,^14, 38^ we showed that primary APL blasts and Lin^−^ HPCs from PR mice were characterized by higher levels of *γ*-H2AX than those detectable in normal human and murine HPCs. Interestingly, high levels of *γ*-H2AX foci were found in human tumors and cultured cells,^39^ during the S phase of the cell cycle, and during apoptosis.^34^ The cell cycle analysis in APL blasts and in PML-RAR*α*-expressing cells seems to exclude the possibility that H2AX phosphorylation depends on the percentage of cells in the S phase or on apoptotic events. Interestingly, PML-RAR*α* expression increases the capability of histone deacetylase inhibitors (HDACi) to induce DNA damage and apoptosis by affecting the expression of genes involved in DNA repair mechanism.^38^ This may explain HDACi selectivity in causing cancer cell death in certain transformed cells.^40^

As *γ*-H2AX represents a biomarker of the DSB, but not a functional component of the DDR,^2, 3, 4^ we further analyzed DSBS sensing using 53BP1, a protein that accumulates within PML-NBs and is involved in DSBs repair.^41, 42^ The time-course analysis of 53BP1 recruitment at the DSBs in *in vitro* and *in vivo* models of APL indicated that the disruption of the PML-NBs slows its recruitment at the DSBs after IR. 53BP1 was nonrandomly associated with PML-NBs and its distribution within the nucleus was dependent on PML-NBs integrity. Furthermore, we observed that in myeloid cells the number of 53BP1 and PML associations follows the DSBs rejoining profile, indicating an association of the two proteins at the DSB foci *in vivo*. Indeed, a defective recruitment of 53BP1 to the damaged sites was observed in PML-RAR*α*-expressing cells, the proper localization being re-established by RA treatment.

Overall, our findings suggest that the expression of the PML-RAR*α* fusion protein, by destroying the PML-NBS integrity, slows down the DSBS rejoining kinetics in human APL blasts and cell lines, and in hematopoietic progenitors from the APL mouse model. This may contribute to the persistence of the higher number of *γ*-H2AX and 53BP1 foci at longer times from IR in APL cells (when compared with myeloid progenitors expressing normal PML protein and PML-NBs), resembling the deficiency in the repair of specific subset of DSBs described in DDR-defective cells.^43^ As PML-NBs are defined as baskets that contain proteins involved in the DSBS response, their disruption may cause a pan-nuclear dispersion of DDR proteins and consequently a defect in the DSBS tethering.^10, 11, 13, 15, 16, 44^

The disruption of the PML-NBs by PML-RAR*α* expression does not increase the chromosomal instability in NB4 cells treated or not with RA and/or IR, although a compromised ability to maintain a genomic stability has been suggested as part of APL pathogenesis.^16^ Most of the somatic events in APL genomes appear to be random background mutations in the hematopoietic cells that acquired the PML-RAR*α* initiating event.^45, 46^ A broad spectrum of acquired and recurrent chromosomal abnormalities, which may act as secondary events and could explain APL leukemogenesis, were reported by high-resolution single-nucleotide polymorphism array (SNP-A) analysis in ~50% matched diagnosis and remission samples from 48 APL cases. These genetic abnormalities were undetectable by conventional cytogenetics.^47^ Almost 90% of these lesions were not exclusive of APL, but recurrent in non-APL acute myeloid leukemias and other hematologic neoplasms.^47, 48, 49, 50^ Notably, mutations present in human leukemias were also identified by the sequencing of an APL mouse genome.^51^ Therefore, the PML-RAR*α* initiating event may be necessary, but not sufficient, to cause APL; additional hits, largely unknown and possibly associated with the HPCs impaired capability to properly repair the DNA lesions,^52^ may occur and ultimately lead to the clonal expansion of leukemic blasts.

PTMs regulate multiple biological functions of PML and of PML-NBS disassembly and rebuilding during the DDR,^21^ and in turn PML-NBs regulate the PTMs of nuclear proteins dependent upon the ATM and ATR kinases.^11, 12, 13, 53^ In this context, our results suggest the existence of a feedback mechanism between PML-NBs and the ATM–NBN–CHK2 axis in the DSBS response. Beside a direct role of these proteins in modulating PML-NB roles in the IR-induced DSBS response,^11^ PML-NBS integrity may be necessary to rapidly activate the DSBS sensing, signaling, and repair. Our results highlight that PML-NBS disruption by PML-RAR*α* strongly affects ATM activation, as well as CHK2 and NBN phosphorylation. This suggests that the PML-NBs integrity is necessary to properly sense DSBs in order to activate ATM and to allow ATM-dependent phosphorylation of proteins localized within PML-NBs.^11, 12, 52^ The delayed recruitment of 53BP1 at the DSBs in PML-RAR*α*-expressing cells, harboring disrupted PML-NBs, together with the impaired increase of RAD51 levels in PR mice, suggest that the HRR mechanism is facilitated by intact PML-NBs *in vivo*.

Data here obtained in human primary APL blasts and in myeloid cells, further confirmed in a preleukemic mouse model of APL, highlighted the relevant role of PML-NBs in coordinating and regulating the DDR. These results further shed light on the events occurring at the onset of APL, suggesting that the expression of PML-RAR*α* disrupts PML-NBs, causes DNA damage, and is responsible for a defect in the early and late steps of the DDR that in turn play a role in the pathogenesis and progression of APL.

## Materials and Methods

### Reagents and antibodies

The complete list of reagents and antibodies is reported in Supplementary Materials and Methods.

### Human samples, cell lines, and cultures

Leukemic cells were isolated from the peripheral blood of three consenting, newly diagnosed APL individuals presenting >80% circulating blasts and classified as M3 or M3v by morphological criteria according to the French–American–British (FAB) classification. The main biologic and clinical features of patients are summarized in Supplementary Table S1. Analysis of the PML-RAR*α* fusion gene was performed by RT-PCR as reported elsewhere.^54^ CD34+ HPCs and mononuclear CD34+ cell fractions were isolated from the peripheral blood of informed healthy donors according to institutional guidelines as previously reported.^55^ Flow cytometric analysis confirmed that >90% of the cells were CD34+.

The human myeloid leukemia cell lines were: the APL-derived NB4 cell line containing the t(15;17)^56^ and the RA-resistant NB4-MR4 subclone;^57^ the human promonocytic U937 cell line either WT (U937/WT) or carrying the PML-RAR*α* sequence under the control of the zinc-inducible MT-I promoter (U937/PR9) or an empty MT1 vector (U937/MT);^58^ the human myeloblastic leukemia cell line HL60 and the RA-resistant HL60-R subclone.^59^ NB4-MR4 and HL60-R resistant cell lines carry mutations abrogating RA-binding capacities of PML-RAR*α* and RAR*α*, respectively. Cell culture conditions and treatments are reported in Supplementary Materials and Methods.

### APL murine model

Experiments were performed using a previously described APL knock-in mouse model^37^ in accordance with the national and international laws and policies. Experimental details are described in Supplementary Materials and Methods.

### Analysis of cell cycle, differentiation, and apoptosis

Cell cycle, cell differentiation, and apoptosis were analyzed with a Dako Galaxy Flow Cytometer (Dako Denmark A/S, Glostrup, Denmark) equipped with a 488 nm laser source and with the FloMax software (version 2.4e; Partec GmbH, Munster, Germany). Hypodiploid peak analysis was performed as previously described.^60^ Cell differentiation was evaluated by direct immunofluorescence staining using fluorochrome-conjugated anti-CD11b antibody.^14, 32^ Details are in Supplementary Materials and Methods.

### Immunofluorescence analysis

Cells were seeded on a glass coverslip using the Shandon Cytospin III (Thermo Fisher Scientific Inc., Waltham, MA, USA), and then fixed, stained, and analyzed as described in Supplementary Materials and Methods.

### Multicolor FISH

The mFISH was performed by hybridizing chromosome spreads with the 21 × mFISH Probe Kit (MetaSystems, Altlussheim, Germany) as described elsewhere.^41^ Karyotyping and cytogenetic analysis of each single chromosome was performed by the ISIS software (MetaSystems, Altlussheim, Germany). Detailed procedures are in Supplementary Materials and Methods.

### Immunoblotting

Protein extraction and western blot analysis were performed by standard procedures reported in Supplementary Materials and Methods.

### Statistical analysis

Results are shown as the mean±S.D. derived minimally from three independent experiments. Statistical significance between means was assessed by Student's *t*-test (GraphPad Software Inc., San Diego, CA, USA). Statistical significance was considered as *P-*values of <0.05.

## Abbreviations

APL: acute promyelocytic leukemia
DDR: DNA damage response
DSB: DNA double-strand break
IR: ionizing radiation
*γ-*H2AX: H2AX phosphorylation at Ser139
HDACi: histone deacetylase inhibitor
HPC: hematopoietic progenitors
HRR: homologous recombination repair
IRIF: ionizing radiation-induced foci
mFISH: multicolor FISH
NHEJ: non-homologous end-joining
PML: promyelocytic leukemia
PML-NB: PML nuclear body
PTM: post-translational modification
RA: all-*trans* retinoic acid
