# Supplementary material for: PML nuclear body disruption impairs DNA double-strand break sensing and repair in APL
Source: Cell Death Dis. 2016 Jul 28;7(7):e2308–. doi: 10.1038/cddis.2016.115 (PMC4973339; doi:10.1038/cddis.2016.115)
Supplement: Supplementary Information [file cddis2016115x1.doc]

**PML nuclear bodies disruption impairs DNA double strand breaks sensing and repair in APL**

**Alessandra di Masi^1,2,*,§^, Domenica Cilli^1,§^, Francesco Berardinelli^1^, Alessandra Talarico^1^, Isabella Pallavicini^3^, Rosa Pennisi^1^, Stefano Leone^1^, Antonio Antoccia^1^, Nelida Ines Noguera^4,5^, Francesco Lo-Coco^4,5^, Paolo Ascenzi^1,2^, Saverio Minucci^3^, Clara Nervi^6^**

^1^ Department of Science, Roma Tre University, Viale Guglielmo Marconi 446, 00146 Rome, Italy

^2^ Istituto Nazionale di Biostrutture e Biosistemi, Viale Medaglie d’Oro 305, 00136 Rome, Italy

^3^ IFOM-IEO Campus, Via Adamello 16, 20139 Milan, Italy

^4^ Department of Biomedicine and Prevention, University of Rome “Tor Vergata”, Italy;

^5^ Neuro-Oncoematology Unit, Santa Lucia Foundation, Rome, Italy

^6^ Department of Medico-Surgical Sciences and Biotechnologies, University of Rome "La Sapienza", Corso della Repubblica 79, 04100 Latina, Italy

**Running title:** PML nuclear bodies and DSB repair in APL

^§^ These Authors contributed equally to this work.

* Corresponding authors: Alessandra di Masi, Department of Science, Roma Tre University, Viale Guglielmo Marconi 446, 00146 Rome, Italy.

E-mail: alessandra.dimasi@uniroma3.it, Tel: +39-06-57333621, Fax: +39-06-57336321.

**Supplemental Material and Methods**

*Reagents and antibodies*

Chemicals were from Sigma-Aldrich (St. Louis, MO). Bradford protein assay was from Bio-Rad (Hercules, CA). Anti-phospho-Ser1981-ATM (10H11.E12), anti-phospho-Thr68-CHK2 (Thr68-R), anti-CHK2 (A-12), anti-H2AX (H-124), anti-phospho-Ser139-H2AX (Ser139), anti-phospho-Ser343-NBN (Ser343-R), anti-PML (H-238), anti-RARα (C-20), anti-RAD51 (H-92), and anti-tubulin (TU-02) antibodies were from Santa Cruz Biotechnology (Santa Cruz, CA). Anti-53BP1 (NB100-305), anti-ATM (2C1(1A1)), and anti-NBN (NBS1-501) antibodies were from Novus Biologicals (Cambridge, UK). Anti-phospho-Ser2056-DNA PK (ab18192) and anti-DNA PK (ab70250) were from Abcam (Cambridge, UK). Anti-phospho-Ser139-H2AX (JBW301) was obtained from Millipore (Billerica, MA). Secondary antibodies were from Immunological Sciences (Rome, Italy). The chemiluminescence reagent for immunoblot analysis was from Thermo Fisher Scientific (Waltham, MA).

*Cell lines and cell cultures*

APL primary blasts and myeloid cell lines were maintained in RPMI 1640 medium (GIBCO Life Technologies, Monza, Italy) supplemented with 10% fetal calf serum (VWR International, Milan, Italy), 100 μg/ml penicillin and streptomycin (VWR International). U937/PR9 cells were induced to express PML-RARα by 100 µM ZnSO_4_-7H_2_O administration. All the cell lines were mycoplasma free. Where indicated, cells were treated with 10 μg/ml cycloheximide (CHX) 0.5h before 1 Gy of X-rays. DSBs were induced using a Gilardoni X-ray apparatus (MGL 300/6-D, 250 kV, 6 mA, Cu filter, dose rate 0.53 Gy/min).

*APL murine model*

The number of animals per group has been maintained to the minimum value to reach statistical significance, *i.e.*, 3 mice/group. Animals were randomly and blinded allocated to treatment groups. C57BL/6 wild-type and PML-RARα knock-in 14-week-old female mice were exposed to the sublethal dose of 5.5 Gy of X-rays, and sacrificed after 0.5, 3, 6, and 24h. Lin^–^ cells were pooled and purified from bone marrow through a Ficoll gradient. Mononucleated cells were enriched for progenitors by depletion of cells presenting myeloid, erythroid, and lymphoid differentiation markers using commercially available reagents (Stem Cell Technologies, Vancouver, BC, Canada).

*Analysis of cell cycle, differentiation and apoptosis*

For cell cycle analysis and apoptosis analysis, 1×10^6^ cells were fixed with 70% cold ethanol and rehydrated with PBS. After centrifugation, pellets were resuspended in PBS containing 0.18 mg/ml propidium iodide (PI) and 0.4 mg/ml DNase-free RNase (type 1-A), incubated for 0.5 h at 37°C, and analysed. Minimums 20.000 total events were acquired in logarithmic scale, and the percentage of the sub G1 population was calculated by drawing an electronic marker. For CD11b analysis, 1×10^6^ cells untreated or exposed to 1µM RA and/or 1 Gy of X-rays, were washed with 1% BSA/PBS and incubated for 30 min on ice with the anti-CD11b PE-conjugated antibody (BD Biosciences, San Jose, CA; dilution 1:50). After that, cells were washed twice with PBS and immunofluorescence intensity was measured. Results were presented as the fluorescence intensity distribution of CD11b membrane expression. Dead cells were omitted from analysis by side scatter electronic gate exclusion.

*Multicolor FISH (mFISH)*

Chromosome spreads were obtained following incubation with calyculin-A (Wako, Osaka, Japan). Spreads of prematurely condensed chromosomes were treated with 75 mM KCl hypotonic solution for 20 min at 37°C, and fixed in Carnoy solution. Cells were dropped onto glass slides and hybridized with the 21X mFISH Probe Kit (MetaSystems, Altlussheim, Germany) as described elsewhere.^41^ Metaphases were captured with the Axio Imager M1 microscope (Carl Zeiss, Oberkochen, Germany). Karyotyping and cytogenetic analysis of each single chromosome were performed by the ISIS software. At least 100 cells for each sample were analyzed in two independent experiments.

*Immunofluorescence analysis*

Cells were fixed in ice-cold methanol for 15 min and in ice-col acetone for 2 min, and blocked in 10% BSA dissolved in PBS (W/V) for 1h at RT. Following incubation with primary antibodies diluted in 1% BSA/PBS over night at 4°C, slides were incubated for 1h at 37°C with 10 µg/ml of Alexa Fluor 488-conjugated goat anti-mouse IgG or Alexa Fluor 610-conjugated goat anti-rabbit antibodies. Confocal analysis was performed using the LCS Leica confocal microscope (Leica Microsystems, Heidelberg, Germany). Quantitative analysis was carried out by counting foci in at least 100 cells/experiment, in three independent experiments.

*Immunoblotting*

Human cells were lysed in 20 mM Tris-HCl pH 8, 137 mM NaCl, 10% glycerol (v/v), 1% NP-40 (v/v), 10 mM EDTA, and protease inhibitors. Mouse Lin^–^ cells were lysed in 8 M urea, 25 mM Tris-HCl pH 6.8, and 10% glycerol. Protein extracts (30 μg) were resolved by SDS-PAGE, and transferred to PVDF (Bio-Rad). Membranes were blocked with 3% BSA/PBS and 0.5% Tween-20 (v/v), probed with the appropriate primary antibodies at 4 °C overnight, and incubated with HRP-conjugated secondary antibodies 1h at RT. Proteins were visualized using the enhanced chemiluminescence detection.

**Supplemental Figure Legends**

**Supplemental Figure S1.** (A) Representative images of the double immunofluorescence analysis of γ-H2AX (Alexa Fluor 488, green fluorophore) and PML (Alexa Fluor 610, red fluorophore) foci in NB4-MR4 cells untreated or treated with 1 μM RA for 72h, and then exposed to IR and fixed after 0.5, 3, 24 and 48h (cell image: bright field; confocal microscopy images, magnification × 63; scale bar: 20 μm). (B) Representative immunoblot analysis of RARα and PML-RARα expression levels in NB4 and NB4-MR4 cells treated or not with 1 μM RA for 48, 72, and 96h. Filters were probed with anti-RARα antibody and tubulin was used as loading control. (C) Percentage of NB4 and NB4-MR4 cells expressing CD11b. Cells either untreated or treated with 1 μM RA for 72h and then irradiated with 1 Gy and fixed after 24 and 48h.

**Supplemental Figure S2.** (A) Percentage of HL60 and HL60-R cells expressing CD11b. Cells were either untreated or treated with 1 μM RA for 72h and then irradiated with 1 Gy and fixed after 24 and 48 h. (B) Quantification of γ-H2AX foci/cell in HL60, HL60-R, and U937/WT cells, untreated or treated with 1 μM RA for 72h and then exposed to IR and fixed at the indicated times. Mean values were derived from the analysis of 100 cells in three independent experiments ± standard deviations. (C) Percentage of DSBs persisting at various times after IR in HL60, HL60-R, and U937/WT cells, taking the mean number of γ-H2AX foci scored at 0.5h after IR as 100%. (D) Quantification of 53BP1 foci/cell, reported as the mean value of 53BP1 foci at each time point after IR in HL60, HL60-R, and U937/WT cells untreated or treated with 1 μM RA for 72h. Error bars represent the standard deviation from the analysis of 100 cells from three independent experiments.

**Supplemental Figure S3.** (A) Double immunofluorescence analysis of pSer1981-ATM (Alexa Fluor 488, green fluorophore) and PML (Alexa Fluor 610, red fluorophore) localizations in U937/WT cells irradiated with 1 Gy and fixed after 0.5 and 3h. (B) Double immunofluorescence analysis of pSer1981-ATM (Alexa Fluor 488, green fluorophore) and pSer343-NBN (Alexa Fluor 610, red fluorophore) localizations in U937/WT cells either untreated or exposed to 100 μM ZnSO_4_, then irradiated with 1 Gy and fixed after 0.5 h and 3h. (Couterstain: DAPI; confocal microscopy images, magnification × 63; scale bar: 20 μm; LCS Leica confocal microscope (Leica Microsystems, Heidelberg, Germany).

**Supplemental Figure S4.** Immunoblot analysis of ATM, NBN and CHK2 phosphorylation in HL60 and HL60-R cells untreated or treated with 1 μM RA for 72h and then exposed to IR and fixed after 0.5, 3, and 24h.

|  | **Supplemental Table S1. Biological and clinical features of the APL patients at diagnosis** | | | | | | | | |
| --- | --- | --- | --- | --- | --- | --- | --- | --- | --- |
|  | | **SEX** | **AGE** | **FAB** | **% of blasts (BM)** | **PML-RARα isoform** | **PML-NBs** | **Sanz risk** | ***FLT3*-ITD status** |
| **APL#1** | | **F** | **62** | **M3** | **80%** | **bcr1** | **disrupted** | **intermediate** | **negative** |
| **APL#2** | | **F** | **54** | **M3** | **85%** | **bcr1** | **disrupted** | **low** | **negative** |
| **APL#3** | | **M** | **32** | **M3v** | **80%** | **bcr3** | **disrupted** | **high** | **positive** |

Patients were classified as M3 or M3-variant according to the French-American-British (FAB) classification.^1^ The so-called "Sanz score" classifies APL patients according to peripheral blood counts into three risk groups: low (WBC ≤10×10^9^/L and platelet count >40×10^9^/L), intermediate (WBC ≤10×10^9^/L and platelet count ≤40×10^9^/L), and high (WBC >10×10^9^/L), thus allowing the design of distinct therapeutic strategies for this disease. ^2^ PML-NBs and *FLT3* status for the ITD are also reported.

Abbreviations: APL, acute promyelocytic leukemia; F, female; M, male; FAB, French-American-British Classification; BM, bone marrow; bcr, breakpoint cluster region; PML-NB, PML nuclear bodies; *FLT3*-ITD, internal tandem duplication (ITD) mutations in the *fms*-*like tyrosine kinase 3* (*FLT3*) gene.

**References**

1. Bennett JM, Catovsky D, Daniel MT, Flandrin G, Galton DA, Gralnick HR, Sultan C. Proposed revised criteria for the classification of acute myeloid leukemia: a report of the French-American-British Cooperative Group. *Ann Intern Med* 1985; **103**: 620-625.
2. Sanz MA, Lo Coco F, Martin G, Avvisati G, Rayón C, Barbui T, Díaz-Mediavilla J, Fioritoni G, González JD, Liso V, Esteve J, Ferrara F, Bolufer P, Bernasconi C, Gonzalez M, Rodeghiero F, Colomer D, Petti MC, Ribera JM, Mandelli F. Definition of relapse risk and role of nonanthracycline drugs for consolidation in patients with acute promyelocytic leukemia: a joint study of the PETHEMA and GIMEMA cooperative groups. *Blood* 2000; **96**: 1247-1253.
