# Supplementary figures and images for: PML nuclear body disruption impairs DNA double-strand break sensing and repair in APL
Source: Cell Death Dis. 2016 Jul 28;7(7):e2308–. doi: 10.1038/cddis.2016.115 (PMC4973339; doi:10.1038/cddis.2016.115)

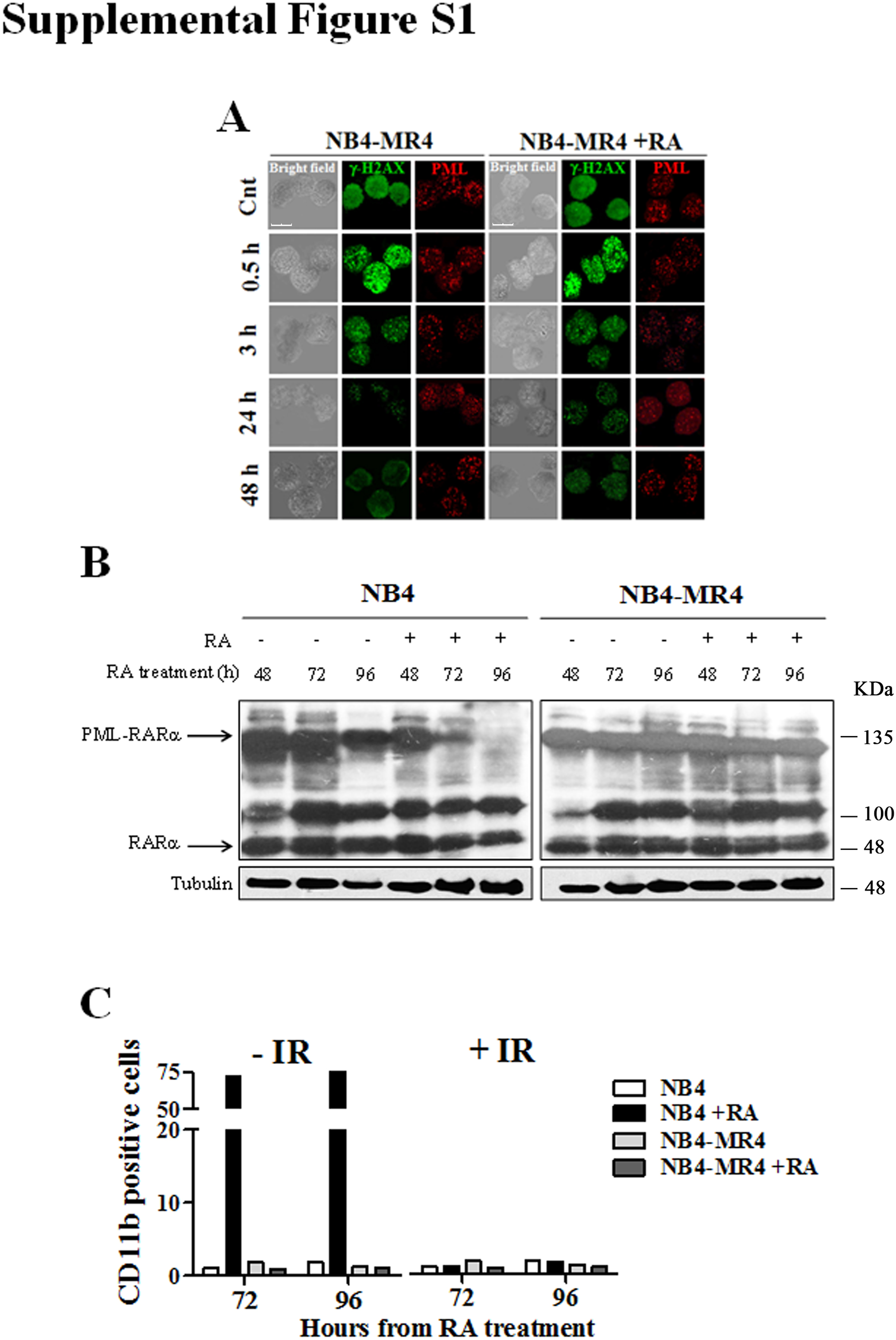

Supplement: Supplementary Figure 1 [file cddis2016115x2.tif]

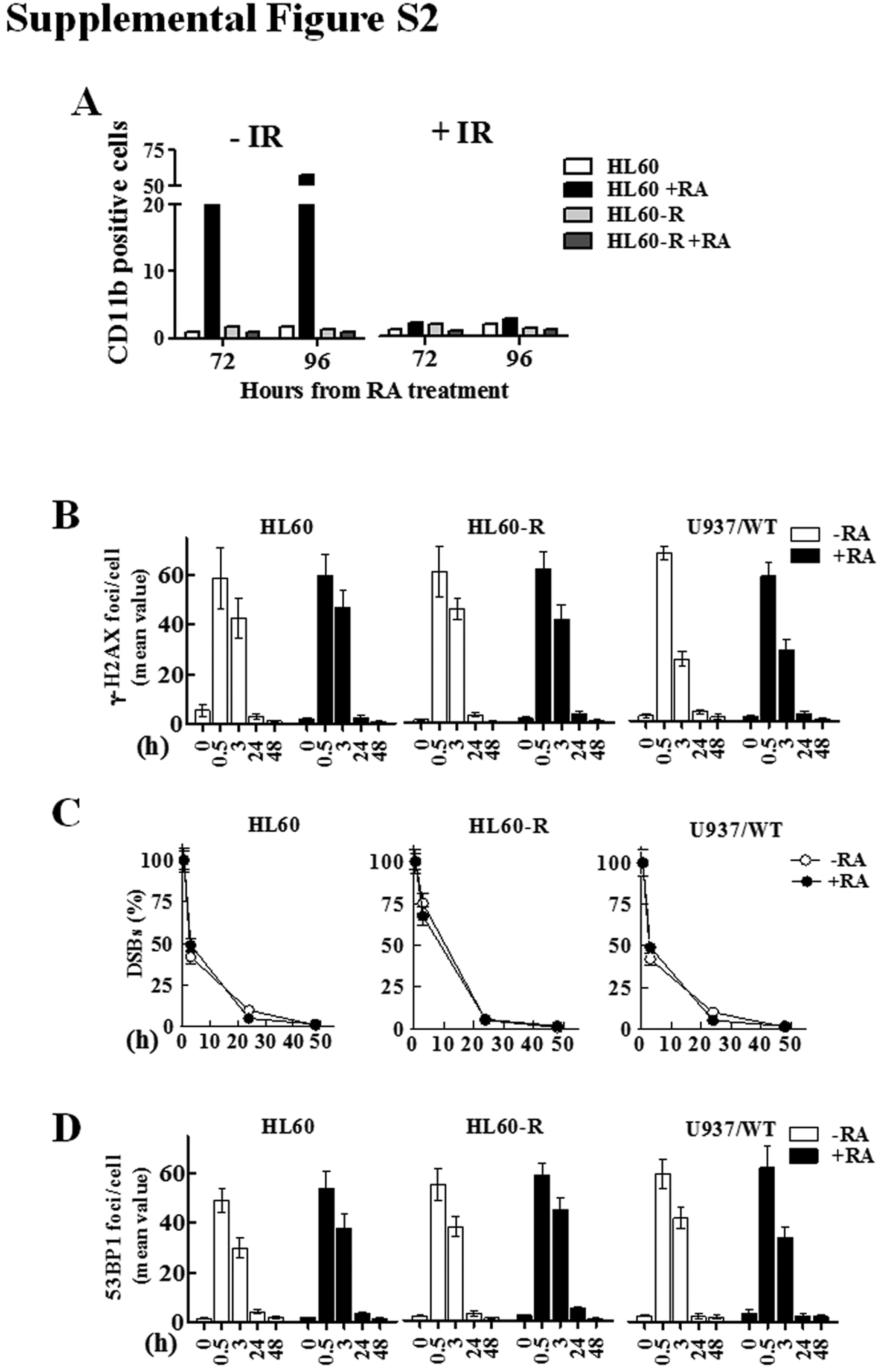

Supplement: Supplementary Figure 2 [file cddis2016115x3.tif]

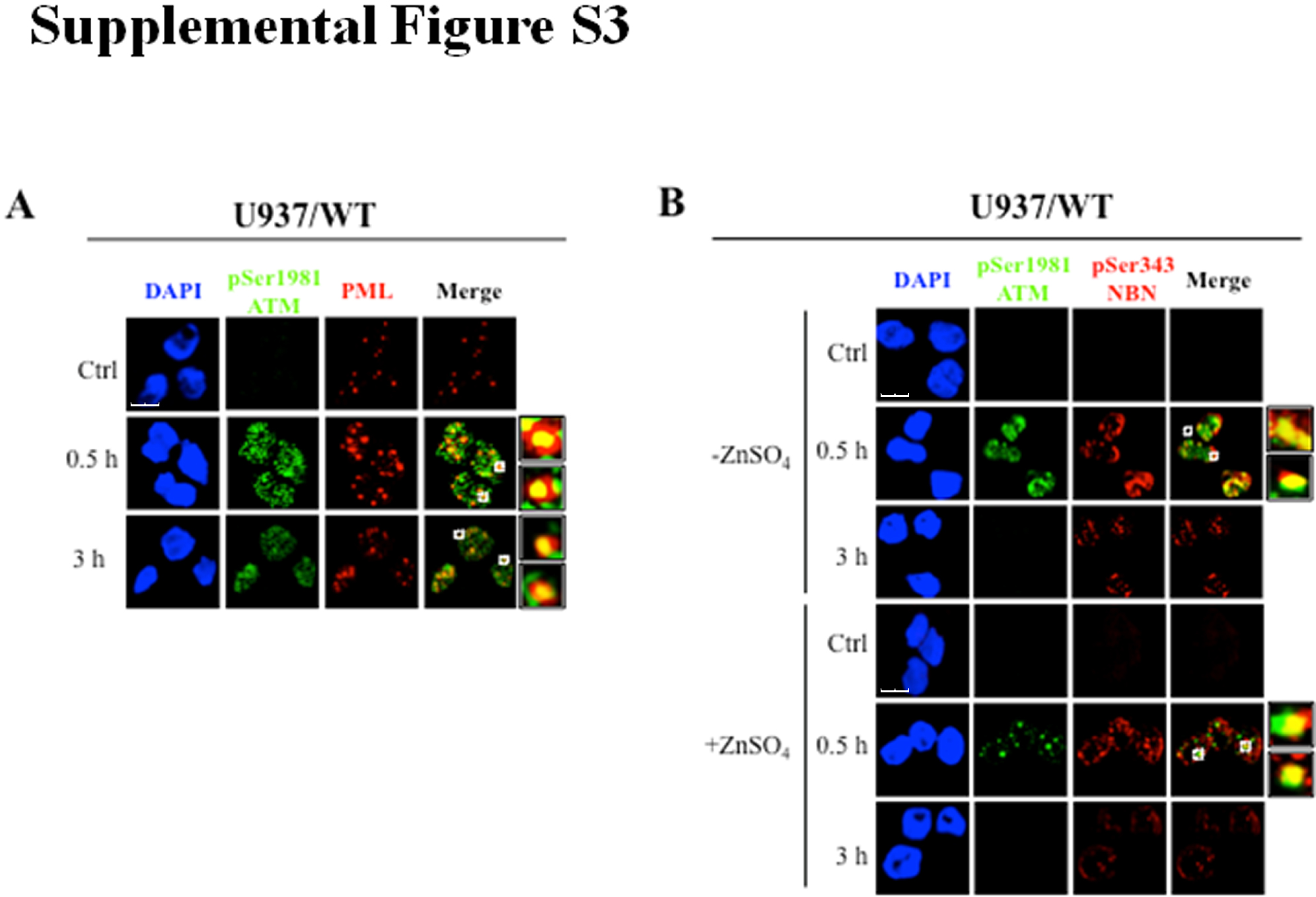

Supplement: Supplementary Figure 3 [file cddis2016115x4.tif]

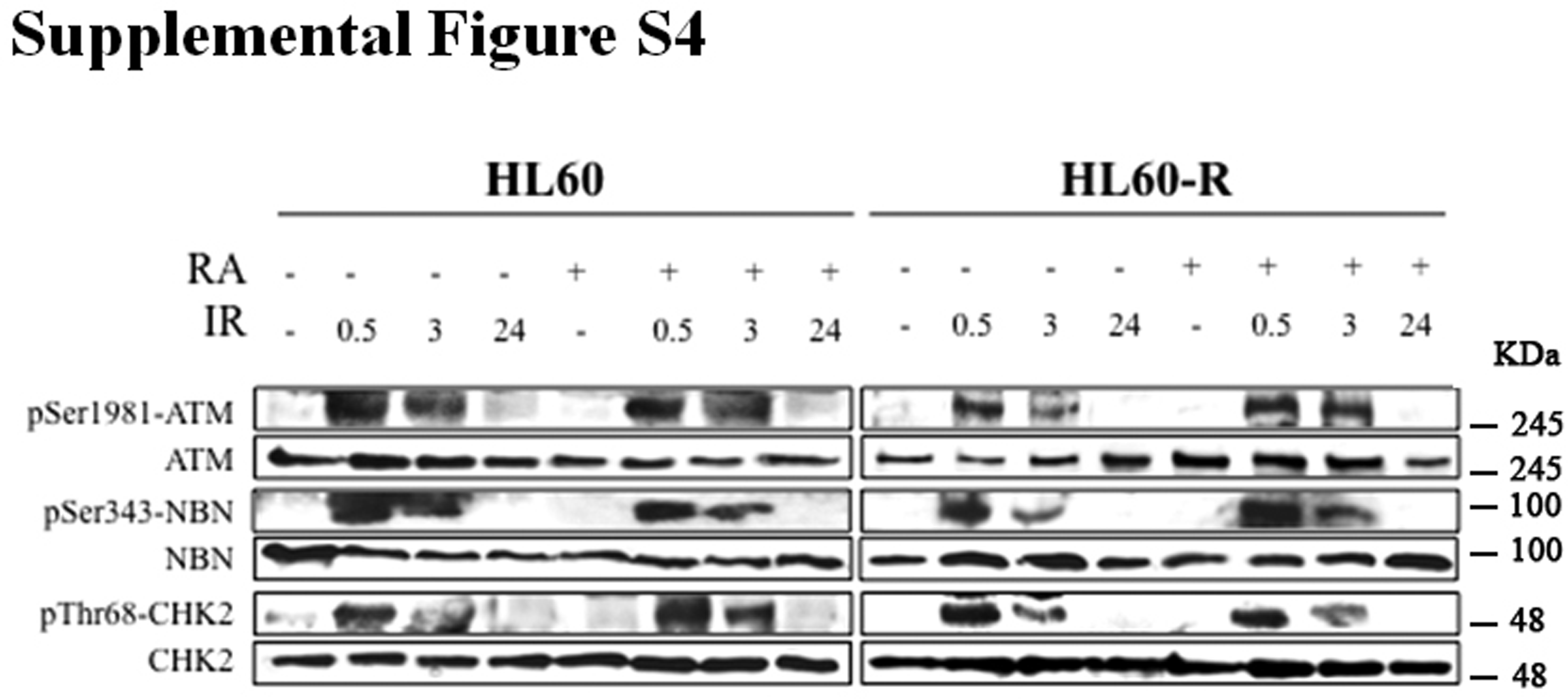

Supplement: Supplementary Figure 4 [file cddis2016115x5.tif]
